# Governance in health workforce: how do we improve on the concept? A network-based, stakeholder-driven approach

**DOI:** 10.1186/s12960-020-00545-0

**Published:** 2021-01-02

**Authors:** Max Ying Hao Lim, Vivian Lin

**Affiliations:** Li Ka Shing Faculty of Medicine, The University of Hong Kong, 21 Sassoon Road, Pokfulam, Hong Kong China

**Keywords:** Health workforce governance, Stakeholder participation, Network governance, Governance conceptualisation, Human resources for health, Health systems strengthening

## Abstract

**Background:**

Health workforce governance has been proposed as key to improving health services delivery, yet few studies have examined the conceptualisation of health workforce governance in detail and exploration in literature remains limited.

**Methods:**

A literature review using PubMed, Google Scholar and grey literature search was conducted to map out the current conceptualisation of health workforce governance. We identified all published literature relating to governance in health workforce since 2000 and analysed them on two fronts: the broad definition of governance, and the operationalisation of broad definition into key dimensions of governance.

**Results:**

Existing literature adopts governance concepts established in health literature and does not adapt understanding to the health workforce context. Definitions are largely quoted from health literature whilst dimensions are focused around the sub-functions of governance which emphasise operationalising governance practices over further conceptualisation. Two sub-functions are identified as essential to the governance process: stakeholder participation and strategic direction.

**Conclusions:**

Although governance in health systems has gained increasing attention, governance in health workforce remains poorly conceptualised in literature. We propose an improved conceptualisation in the form of a stakeholder-driven network governance model with the national government as a strong steward against vested stakeholder interests. Further research is needed to explore and develop on the conceptual thinking behind health workforce governance.

## Introduction

It is well established that key systemic issues in health workforce (quantitative shortage [1], skills-mix, distribution imbalance, and more) can only be tackled through a cohesive and strategic governance approach, and improvements in health workforce performance deliver key health outcome gains in indicators such as under-five mortality [2-4]. Yet governance of health workforce remains relatively neglected [5] and described as the ‘elephant in the room’ [6]. Given the centrality of the health workforce for health service delivery, understanding the ‘governance’ question in health workforce is key to delivering people-centred care [7] and improving overall health systems performance.

Discussions on governance and health have gained increasing attention [8-10] since 2000 when the World Health Organization (WHO) first defined ‘stewardship’ as one of the four key functions underpinning a performing health system [11]. However, within health workforce literature, the operationalisation and conceptualisation of governance remains limited.

Coordinating and implementing policies for the health workforce may require governance which is divergent from a top-down hierarchical governance process, not least because power is shared amongst a diverse array of stakeholders which limits the authority of governments and public institutions. Thus, renewed and fresh considerations regarding health workforce governance is required in order to ensure effective and responsive human resources for health (HRH) stewardship and policymaking in areas such as health workforce assessment, planning and feedback-monitoring [12]. An improved understanding and conceptualisation will empower governance-based improvements which will drive adaptability, resiliency and efficiency within the health system, ultimately spearheading progress towards people-centred care and universal health coverage.

### Purpose and rationale

This review aims to outline the current conceptualisation of governance within health workforce literature. Governance is a process with different implications for different actors; however, considerations towards governance will be focused on the national level due to the stewardship responsibilities bestowed on national entities in advancing global health initiatives (e.g. universal health coverage). Up to now, no such review is known to have be published in existing literature.

Findings are modelled after Barbazza and Tello’s previous work in conceptualising and bringing applicability of governance to a health system setting [13]. As governance is fundamentally a systems-based process of which core characteristics can be determined, we will focus our findings on the *definition* of governance (an objective description), and the *dimensions* of governance (an operational characterisation) as they apply to the health workforce. A discussion will begin with a critique of the current conceptualisation of governance followed by suggestions on possible ways to move forward.

## Methods

A literature review was conducted to rapidly aggregate literature findings up until July 2020. Inclusion criteria were limited to all literature pertaining to health workforce and governance since the year 2000 (when ‘stewardship’ was first established by the WHO). Sources must explicitly discuss governance in reference to health workforce, whilst literature which marginally mentions governance will only be considered more generally. Search terms include a variety of combinations of key terms including ‘governance’, ‘stewardship’ and ‘leadership’ in addition to ‘health workforce’ and ‘human resources for health’. Searches for published literature were conducted through PubMed and Google Scholar, with additional searches for grey literature through Google Search.

The main perspective adopted towards governance will be from the national public level; as the state government is bestowed with powers in legislation, financing and leadership, it acts as an ideal point of intervention for further discussion. This does not necessarily restrict literature findings as most literature already present national or supranational perspectives, or derive from international organisations that routinely deal with national governments as primary ‘nodes’ for domestic policymaking.

## Findings

In total, 28 publications were identified. This included 12 case studies referencing domestic or regional governance in health workforce, either by outlining the general landscape and challenges (10) [14-23], or by promoting new governance-based approaches (2) [24, 25]. Of the remaining sources, seven were broader systematic reviews, literature reviews, scoping studies, case studies or books that were able to consider governance more generally within health workforce [5, 26-31], two were region-wide examinations of HRH units within countries [32, 33], and six were editorials, research articles or other grey literature [7, 34-38]. One publication was considered exceptionally due to its discussion of health workforce governance from a public administration perspective [39]. Six publications (including 1 editorial) were extracted from the ‘Health Workforce Governance in Europe’ special issue of Health Policy (Volume 119, Issue 12, December 2015).

### What is the *definition* of governance?

Within health literature, there is already extensive writing on the conceptualisation and definition of governance with broad consensus that governance represents the *efforts and processes* [13] through which priorities are achieved. ‘Stewardship’ further adopts the additional element of *direction*, i.e. in defining an overarching vision, goal and ‘playing field’.

As for health workforce literature, Table 1 outlines the scope of definitions for governance. Most sources make reference to established definitions in health literature with Barbazza and Tello’s [13] and Brinkerhoff and Bossert’s [40] definitions being the most popular. This characterisation of governance is process-oriented: it seeks to describe a set of *processes* through which rules and responsibilities are distributed amongst different actors to achieve goals. Brinkerhoff and Bossert [40] further describe governance with a political dimension, adding specific reference to authority, power and decision-making in shaping policy.

**Table 1:** A selected list of definitions of governance from health workforce literature

Author (alphabetical)	Definition of governance	Orientation
Adeloye et al. [14]	The administrative umbrella of the health system primarily concerned with policymaker- or government-led steering and rule-making functions targeted at achieving national health policy objectives for effective delivery of health services and attainment of universal health coverage	Outcome-oriented
Barbazzaa et al. [27]	[Bringing] better alignment between the day-to-day functioning of services delivery and the health system … as a minimum, we consider these processes to include setting priorities for the system's direction, organizing for action across actors, and measuring and feeding-back on performance	Mixed
Dieleman et al. [5]	The rules that distribute roles and responsibilities among government, providers and beneficiaries and that shape the interactions among them. Governance encompasses authority, power, and decision-making in the institutional arenas of civil society, politics, policy, and public administration	Process-oriented
Gallagher et al. [16]	[Ensuring] that an organisation or partnership fulfils its overall purpose, achieves its intended outcomes for citizens and service users, and operates in an effective, efficient and ethical manner	Outcome-oriented
Hastings et al. [28]	A whole range of structures and processes through which policies (formal and informal) are enacted to achieve goals, including legislation, regulation and oversight, accountability structures, incentives, and policies to set and maintain strategic direction. In the context of health systems, governance has been characterized as a set of tasks and functions largely established to carry out health ministry goals—essentially driving the direction, type, and accountability of service delivery to improve health system performance	Mixed
Hazarika [7]	Rules (both formal and informal) for collective action and decision-making among government, providers and beneficiaries that also shape the interactions among them	Process-oriented
Kaplan et al. [26]	The set of rules that define the responsibilities of health system actors, how they operate, and how they relate to one another	Process-oriented
Kwamie et al. [17]	The formal and informal rules which guide behavior	Process-oriented
Manafi et al. [20]	Ensuring that strategic policy frameworks exist and are combined with effective oversight, coalition building, regulation, attention to system design, and accountability	Outcome-oriented
Milicevica et al. [18]	Multi-sector efforts, complex mechanisms and procedures to exercise and mediate the participation of different groups’ rights and interests. Broadly defined, governance shapes the roles, power and interactions among government, providers and beneficiaries	Process-oriented
Rees [19]	The collection of mechanisms, structures, processes and influences for a system’s oversight, policies, planning and accountability	Process-oriented
World Health Organization [32]	The ability of individuals, organizations or systems to perform the functions for HRH development effectively, efficiently and sustainably	Outcome-oriented

There is an alternative, if not complementary definition that defines governance more narrowly as outcome-oriented: it describes the administrative and policymaking tasks required to align policies with health system objectives and to fulfil its overall purpose. This sees governance as a tool for alignment towards set outcomes such as national health objectives and is similar to WHO’s 2007 definition [41] in describing governance through a policy-focused lens. In this characterisation, governance is defined by its ability to achieve intended outcomes and the tasks and processes necessary to do so.

These two characterisations are not competing and should be seen as mutually complementary in describing different perspectives towards governance. Brinkerhoff and Bossert [40] also acknowledge the outcome orientation in further clarifying the delineation of rules for different institutional arenas in order to achieve health sector objectives.

Some sources use the *salient characteristics* of governance (i.e. normative judgments) as a proxy to define, characterise and measure governance. A subset of papers also emphasise governance alongside *government*, with the notion that governance is an intrinsic responsibility of government policymaking; for example, ‘administrative and ‘policymaker’ functions in governance imply to the primary role of the government in driving governance-based changes. This viewpoint sees the government as an end-all organisation through which policies are implemented, of which the implementation can be ‘improved’ through focusing on ‘good’ governance practices—with this entire process being vaguely characterised as ‘governance’.

In summary, the conceptualisation of governance in health workforce literature is either *process driven, outcome focused* or both. Much of the literature extrapolates the concept of governance ad hoc from health governance; however, apart from the work of Kuhlmann et al. [24, 25] and more recently Teter [39], there has been little adaptation or tailoring of governance perspectives towards the specific characteristics and challenges within the health workforce.

### What are the key *dimensions* of governance?

Barbazza and Tello define dimensions as the ‘components, elements, principles or attributes’ of governance—in other words, the compositional makeup of the characterisation of governance. Using the previous effort where the dimensions of health governance were mapped into three categories (fundamental values, sub-functions and outcomes), we adopt the same framework but instead focus on health workforce literature: this is outlined in Table 2. Whilst this framework does not represent a complete nor comprehensive depiction of the dimensions of governance, we see it as broadly accurate in delineating the main dimensions of governance.

**Table 2:** Dimensions of governance across a selection of health workforce literature. Adapted from Barbazza and Tello [13]

Dimensions of governance	Dieleman et al. (2011) [5]	WHO (2012) [32]	Kaplan et al. (2013) [26]	Hastings et al. (2014) [28]	Kuhlmann et al. (2015) [24]	Milicevic et al. (2015) [18]	Vicarelli et al. (2015) [15]	Adeloye et al. (2017) [14]	Manafi et al. (2019) [20]
**Fundamental values**									
Control of corruption	✓								
Democracy									
Human rights	✓								
Ethics and integrity									✓
Conflict preservation									
Public good									
Rule of law	✓								✓
**Sub-functions**									
Accountability	✓		✓		✓	✓		✓	✓
Partnerships	✓	✓			✓			✓	
Formulating policy/strategic direction	**✓**	**✓**	**✓**	**✓**	**✓**	**✓**	**✓**	**✓**	**✓**
Generating information/intelligence	✓	✓	✓		✓	✓	✓		✓
Organisational adequacy/system design		✓		✓	✓		✓	✓	
Participation and consensus	**✓**	**✓**	**✓**	**✓**	**✓**	**✓**	**✓**	**✓**	**✓**
Regulation	✓				✓				
Transparency			✓		✓			✓	✓
**Outcomes**									
Effectiveness	✓	✓							✓
Efficiency	✓	✓	✓					✓	✓
Equity	✓		✓			✓			✓
Quality		✓		✓		✓	✓		
Responsiveness			✓	✓			✓	✓	✓
Sustainability		✓							
Financial and social risk protection									
Improved health								✓	

✓ represents dimensions which are included

On first glance, there appears to be large variability in the specific dimensions used to characterise governance. However, we note the clustering of dimensions around the *sub-functions* of governance which describe the ‘actionable processes [of governance] for which the system's steward has oversight’ [13], and away from more normative descriptors. One explanation for this is that any normative characterisation of ‘good’ governance remains a highly subjective effort often linked with political perspectives on ideas such as the rule of law or human rights. Thus, any attempt at normative characterisation risks steering the debate to a political realm which is often out of scope for most health workforce discussions.

If, however, we focus on the *sub-functions* of governance, would there be a difference in characterisation of governance between general health literature and health workforce literature? Dieleman et al. notes that within health sector governance, management issues are often highlighted, but less attention is given to stakeholder factors and political considerations [5]. However, our findings show that within health workforce literature, *stakeholder participation* is instead underscored as an essential component of the governance process—alongside strategic direction, they constitute the only two sub-functions that are consistently mentioned when discussing governance of the health workforce. Beyond this, the scale and scope of dimensions do not show general consensus or focus.

Whilst there are several caveats to this descriptive effort—for example, many dimensions may remain implicitly assumed and thus not explicitly stated in literature—we can broadly note that discussions of governance are preferred on a *functional* lens where ‘operationalising the function of governance itself’ [13] is seen as a primary motivator. The two sub-functions of stakeholder participation and strategic direction are additionally seen as essential in enabling governance improvements in the health workforce.

## Discussion

The majority of health workforce literature circumvents the governance debate by adapting existing concepts of governance in health literature. This is understandable on two grounds: (1) there remains complex political issues intrinsic to the consideration of governance [10] which is beyond the scope of technocratic discussions, and (2) broader concepts of governance are largely seen as sufficient in describing governance issues within a health workforce.

However, "rules of the game are needed but […] there are games in the rules" [37]; we cannot talk about governance in health workforce without discussing how politics, power arrangements and stakeholder interests influence governance. Firstly (and most importantly), governance is rarely within the sole domain of governments; even if governments do exert significant influence, they are subject to checks and balances from powerful stakeholders such as health professional bodies with vested interests and significant resources [34]. Power differences also exist between different stakeholders (e.g. physicians often exert dominance over other health professionals [42]). This limits the effectiveness of traditional top-down hierarchical governance (or command and control), a key example being the self-governing autonomy of professional bodies on issues such as health professional accreditation. Thus, policy discussions are structurally entrenched within a network–web system of various heterogenous stakeholders within and outside the health sector [43].

Meanwhile, an increase in complexity of governing institutions and issues [24] means that regulatory power is often shifted subnationally and/or transnationally away from the direct control of centralised authorities [30]. Priorities in setting health workforce policies are further influenced by interlinked factors such as fiscal space, current economic policy, employment practices and health workforce resistance to change [33], whilst interests and objectives beyond health—from national sovereignty issues to political manoeuvring alongside external stakeholders in education and training and financing—further muddle the picture. This inevitably leads to challenges in implementing and operationalising HRH policies [44].

We believe that a general characterisation of health governance is insufficient when dealing with governance of the health workforce. We also argue against using ad hoc normative descriptors of governance (i.e. a list of characteristics of ‘good’ governance) before a sufficient conceptualisation of governance within health workforce is established that takes into account the specific, intractable challenges unique to the field. The fundamentally political nature of health workforce issues [45] should not be seen as a *barrier* towards discussing governance issues but rather an opportunity for improving the conceptualisation of governance towards a health workforce context.

### Governance in health workforce: network-based, bottom-up and stakeholder driven

Instead of viewing governance at the domestic level as a public policymaking affair effected primarily in a top-down fashion, an improved conceptualisation of health workforce governance sees the governance process as network-based and stakeholder-driven. Teter previously argued for problem-driven collaborative governance in health workforce which emphasises stakeholder engagement and accountability through robust feedback loops [39]. In this frame, governance refers not only to the *processes* through which responsibilities are distributed amongst different actors, but also the *relationships and connections* within a complex web of interlinked stakeholders wishing to influence the processes of governance.

This characterisation seeks to clarify the distinction between governance and govern*ment*. We believe these are two distinct concepts and that the government is by virtue no more intrinsic within governance than any other stakeholder. Of course, governments often do (and *should*) play a stewardship role in directing policymaking to meet global health objectives; however, this is not within the inherent conceptualisation of governance and assumes erroneously that any government has absolute authority in policymaking. Kuhlmann and Larsen have previously noted multi-level governance as an alternative framework eschewing the traditional hierarchical basis of governance for multi-level, content based governance in which a central government has relatively reduced direct authority [24]; they note that ‘…governance shifts the regulatory power from the “government” to more plural tiers of governance and strengthens operational governance on the levels of organizations and professional groups.’

If the government should be seen as distinct from (but still highly involved with) the governance process, redefining the government’s role is required. Some salient characteristics of this effort are listed below:

1. Government policymaking which acknowledges the fragmentation of formal and informal power and actively navigates through the stakeholder landscape, instead of reasserting power under a central authority;
2. A deep understanding and respect of the complex network of *interconnections* between stakeholders as well as the close-knit, complex relationships of vested interests that underpin governance, stewardship and policy-setting;
3. A proactive willingness to collaborate and negotiate policy decisions with stakeholders, instead of top-down unilateral implementation of policies;
4. An emphasis towards bottom-up consensus-building policymaking that encourages buy-in, trust and collaboration between stakeholders and the government;
5. A keen focus towards achieving health system objectives whilst encouraging stakeholder influence and input within the governance process;
6. Stewardship against vested interests through institutional and regulatory reform where necessary should stakeholder consensus be unachievable.

This conceptualisation simultaneously acknowledges the increasingly crowded stakeholder landscape that dominates the health workforce discussion and empowers the government to seek solutions which include bottom-up stakeholder participation and consensus-building within the policymaking process. One particular model of this approach would be multi-level governance as described by Kuhlmann and Larsen [24] in which—recognising the decentralisation of power to various levels of stakeholders, from subnational to transnational—the focus shifts towards the *interconnections* between stakeholders.

Key stakeholders within the governance process include health professional groups, financiers, regulatory bodies, professional educational institutions and health provider organisations, all with differing interests and requiring strategic stewardship from the government in order to align policymaking focus towards global health objectives. In addition, within government there is also a requirement to collaborate with other intra-governmental ministries, most principally the Ministry of Education (which dictates health professional training and broader policies relating to public and private institutions in education and training) and the Ministry of Finance (which dictates health financing). These also represent *stakeholders* which answer to different constituencies and have different mandates. Therefore, the Ministry of Health must help guide stewardship during the governance process and ensure political commitment from the highest levels of policymaking power.

Emphasis on a stakeholder-driven process should also drive governments to be *context-specific* in governance strategies. This is because stakeholder positions may be similar across countries in the same region, but their powers and roles vary depending on circumstances that are not easily observable [30]. While this makes it harder to promote particular governance strategies between countries (as normative ‘good’ governance practices do very nicely!), we believe the governance *process* should instead be the focal point of intervention rather than the specific policy positions. For example, in a country with a self-regulated professional nursing body which controls professional registration, it is necessary to consolidate stakeholder involvement before seeking any policy changes regarding nursing membership; in another country without self-regulated professional groups, deep stakeholder participation may still be highly desirable due to extensive stakeholder influence. In both cases, the objective may be professional licensing reforms; however, the governance process is adaptive and flexible to stakeholder demands. Pushing for universal governance reforms on ‘equity’ or ‘transparency’ is tone-deaf to domestic policymaking that should also be responsive and adaptive to local circumstance, derived after acquiring a deep and thorough understanding of stakeholder dynamics and interests.

Traditionally, a network governance-based approach has rarely been discussed within health literature and remains a concept developed from business and public policy contexts [39]. We advocate for a ‘brokered-network’ model where the government still retains key ‘broker’ functions (most notably in legislation, public financing and stewardship) whilst other responsibilities are devolved to stakeholders [46]. This model emphasises trust and goal consensus as critical components in the governance process.

### Stewardship in a stakeholder-driven network governance model

It is important to note that in pursuing a network-based, stakeholder approach, the government must continue to sustain stewardship over the health workforce. Stakeholder involvement in the governance process—if appropriately structured—enables capacity-building towards an integrated health workforce [34], whilst bottom-up professional participation can be a catalyst for beneficial policy change. However, if the government relinquishes the fundamental responsibilities of a steward in pursuing stakeholder-driven governance, i.e. in failing to define a vision and set the ‘rules of the game’ in policymaking, governance will be driven by unequal stakeholder interests quasi-independent from health system objectives. For example, professional bodies often initiate salary-related reforms whilst blocking labour market structural changes such as skills-mix or task shifting reforms [30] regardless of overall health workforce issues.

With key steward functions in legislation, public institutional arrangements, financing (and potentially a fourth, informal influence in leadership), the government is ideally suited to act as a ‘broker’ in providing stewardship towards health objectives. We are not proposing that the government abandon their stewardship role through stakeholder participation; rather, we propose that stakeholder expertise is hugely beneficial in the governance process and thus stewardship should be towards governance practices that involve bottom-up stakeholder input.

What would this look like in practice? One possibility could include stakeholder-driven governance deliberately weighted to ‘steward’ against dominant stakeholder influence. Kuhlmann et al. have described a German regional health workforce monitor which counterbalances against the influence of one particularly strong stakeholder group (doctors) by excluding them from the governance process, thus directly connecting smaller diverse stakeholders with policymaking and ‘disrupting a cycle of ineffective health workforce policy’ [25]. Another possibility could include strong top-down leadership, seen when the Ethiopian government increased the production and deployment of doctors to fill workforce gaps despite resistance from professional bodies and medical schools [30]. Potential stewardship approaches should be highly contextual and formed after a thorough understanding of the local health workforce condition.

A bottom-up governance approach can only be successfully pursued with the government firmly as a steward to counterbalance unequal stakeholder interests and direct policymaking towards global health objectives. As potentially the only participant whose interests are most aligned with a health-for-all approach, governments are also the only actors capable of utilising institutional arrangements to negotiate the unequal power dynamics between stakeholders. Thus, governments will have the unenviable task of balancing between driving stakeholder consensus and fulfilling a ‘stewardship’ role—a fundamentally political affair.

### Implications for key players: what does it look like in practice?

In visualising stakeholder-driven governance, we delineate key implications according to the perspectives of various actors in the governance process. Mitchell and Bossert have previously outlined the relative stakeholder positions and interest clashes in a health workforce reform context [30] which serves as a useful complement to the following conceptualisation.

#### Leaders (elected officials)

##### Power: very high

Leaders at the highest level of public office drive the political support necessary towards stakeholder-driven governance reforms both internally (within governmental bodies) and externally (with stakeholder interests). Establishing a strong vision aligned with community interests and global health mandates alongside policymaking which embraces stakeholder involvement is perhaps the most critical link in fostering reform within sectors that may previously be ‘silo-ed’ with little inter-disciplinary collaboration.

#### Government health officials

##### Power: high

Health officials at the national level are responsible for formulating policies towards broader health system objectives with the additional emphasis on encouraging stakeholder participation, establishing a conducive environment for communication and consensus-building, and enabling continual feedback-ing such that policymaking is reflective of stakeholder input. They act as a leader both within government and with external stakeholders in coordinating stakeholder concerns and priorities towards national health objectives.

#### Other governmental ministries

##### Power: medium to high

Other ministries involved in components of health workforce policy must collaborate widely and actively with the Ministry of Health in identifying common issues and feasible solutions in collaboration. The goal is to develop coherent national policies which do not contradict and antagonise other existing policies and priorities within government.

#### Health stakeholders (professional bodies, health facilities)

##### Power: medium to high

Stakeholders responsible for health service delivery should participate actively in the governance process and communicate across disciplines. The focus is on fostering *interconnections* between different stakeholders and with governmental agencies in an organic and bottom-up manner—connections that are both synergistic and functionally relevant help develop resiliency and support in HRH policymaking.

#### Community stakeholders (non-governmental organisations, citizen’s interest groups, etc.)

##### Power: low

It is essential to include community stakeholders directly within the governance discourse in order to counterbalance professional self-interests in the policymaking process. Increasing the diversity of the policy discussion will also ease the alignment of objectives towards national policy targets (which should ideally be community oriented).

## Conclusion

Whilst governance within the context of health systems has gained increasing attention [47], the influence of governance has *not been emphasised enough* in the HRH crisis [6] which we believe is a result of inadequate conceptualisation. The stakes are high: the COVID-19 crisis has exposed how dependent our health systems are on a resilient, well-performing health workforce, which is itself dependent on strong stewardship and governance.

We clarify that governance is not simply an ad hoc consideration in improving the implementation of health workforce policies but a distinct process through which networks of conflicting stakeholder interests influence the formation and implementation of policy decisions. Improving the conceptualisation of health workforce governance deepens our understanding of *health systems* governance, enables the operationalisation of governance policies that improve health workforce performance and ultimately delivers health gains on the path towards universal health coverage and health systems strengthening.

The two key elements of this approach involve the *network-based* dimension and the *stakeholder-driven* dimension: network-based places the emphasis of governance on the *connections* between stakeholders, whilst stakeholder-driven enshrines the bottom-up participatory approach that improves health systems performance and builds consensus. The government still maintains an intractable role in stewardship; however, the focus is on encouraging structures that bring stakeholders together and in fostering bottom-up outputs whilst balancing power dynamics and vested stakeholder interests. We believe this is an improved conceptualisation of governance in health workforce and reflective of how governance can better influence HRH policymaking.

We note this review is limited to secondary sources with no primary data collection or first-hand case studies. We therefore suggest further research into case studies of governance practices across regions and reform efforts aligned with stakeholder-driven network governance, and government-stakeholder interactions where particular groups exercise dominant influence. It is also pertinent to explore the health workforce issues exposed by the COVID-19 pandemic and what role governance must play in future health workforce and health systems strengthening. Expanding the literature will ultimately drive improvements in the conceptualisation and operationalisation of governance in health workforce and, in turn, deliver on the essential attributes of a well-functioning health system towards universal health coverage—quality, efficiency, equity, accountability and resilience.

## Data Availability

The datasets used and/or analysed during the current study are available from the corresponding author on reasonable request.

## Abbreviations

HRH: Human resources for health
WHO: World Health Organization
